# Low-Temperature Carbonized Elastomer-Based Composites Filled with Silicon Carbide

**DOI:** 10.3390/polym12112669

**Published:** 2020-11-12

**Authors:** Andrey A. Stepashkin, Semen D. Ignatyev, Dilyus I. Chukov, Victor V. Tcherdyntsev, Sergey D. Kaloshkin, Elena V. Medvedeva

**Affiliations:** 1 Laboratory of Functional Polymer Materials, National University of Science and Technology “MISIS”, Leninskii prosp, 4, 119049 Moscow, Russia; a.stepashkin@misis.ru (A.A.S.); ignatyev.s.11@gmail.com (S.D.I.); dil_chukov@mail.ru (D.I.C.); kaloshkin@misis.ru (S.D.K.); 2 Institute of Electrophysics, Ural Branch, Russian Academy of Science, Amudsena str., 106, 620016 Yekaterinburg, Russia; lena_p@bk.ru

**Keywords:** composites, rubbers, carbonization, thermal destruction, silicon carbide, thermal conductivity

## Abstract

Thermally stable composites obtained by the low-temperature carbonization of an elastomeric matrix filled with hard dispersed silicon carbide particles were obtained and investigated. Evolution of the microstructure and of mechanical and thermal characteristics of composites during thermal degradation and carbonization processes in a wide range of filling from 0 to 450 parts per hundred rubber was studied. For highly filled composites, the compressive strength values were found to be more than 200 MPa; Young’s modulus was more than 15 GPa. The thermal conductivity coefficient of composites was up to 1.6 W/(m·K), and this magnitude varied slightly in the temperature range of 25–300 °C. Coupled with the high thermal stability of the composites, the observed properties make it possible to consider using such composites as strained friction units instead of reinforced polymers.

## 1. Introduction

The use of reinforced polymer materials makes it possible to create structures with high weight efficiency and optimal combinations of mechanical, antifriction and thermal properties, which allows them to replace traditional metal and ceramic materials successfully. Some of the factors limiting the use of polymers and polymer based composites are low operating temperatures due to low melting points and/or low temperature of the beginning of intense thermal degradation of polymers, and low thermal conductivity, which for a most of polymers lies within 0.05–0.35 W/m·K [1,2,3].

The thermal conductivity of polymers can be increased by creating heat-conducting structures based on fillers with high thermal conductivity, such as carbon nanotubes [4], graphene [5], graphite [6], hexagonal boron nitride (BN) [7], aluminum nitride (AlN) [8], alumina (Al_2_O_3_) [9], silicon carbide (SiC) [10], etc. The percolation threshold in such compositions, at which a significant increase in thermal conductivity occurs, usually lies at filling degrees of above 20 vol.% [11]. However, at such high filling degrees, the mechanical characteristics of the materials usually decrease drastically [12]. Therefore, elaboration of polymer composites with both high thermal conductivity and high strength characteristics is an urgent task.

Heat resistance and the operating temperatures of polymer-based composites are limited by the heat resistance of matrix polymer. Carbon matrices obtained by pyrolysis of various polymer binders [13,14,15], coal tar [16,17,18] and petroleum [19,20,21] pitches have exceptionally high heat resistance. A direct approach in getting carbon matrices via classical carbonization is the rapid implementation of the pyrolysis process, which ends with the formation of char at temperatures of about 500–550 °C, followed by the formation of a carbon matrix at temperatures of 900–1700 °C. It is known that thermal degradation results in an increase in the given polymer material’s thermal stability; however, at the same time, thermal degradation results in a decrease in the strength and deformation characteristics of materials [22,23,24].

Recently, we presented an alternative approach, consigning in the formation of a heat-resistant matrix during thermal degradation via low rate heating. Heating regimes in this case are selected to make the rates of consolidation processes (i.e., rate of cross-linking process and the rate of depolymerization reactions chains termination) higher than the rate of the elastomeric binder’s thermo-oxidative destruction. Volatile pyrolysis products such as N_2_, NO_2_, CO and bound water are removed from the material during heating, and the amount of carbon in the remaining material increases [25,26]. The materials obtained show both high thermal conductivity and good strength characteristics in a wide range of filling amounts. In present study we used this approach to create highly filled composites reinforced with silicon carbide, which is widely used as filler for polymer composites with advanced thermal conductivity [10,12,27,28].

Nitrile butadiene rubber (NBR) is one of the most widespread elastomers, characterized by low cost, high resistance to oil products and high resistance to thermal oxidative degradation. Due to its advanced properties, NBR is widely used as a matrix material in composites reinforced with carbon black [29,30], graphene [31], graphene oxide [32,33], SiO_2_ [34], Al_2_O_3_ [32] and other inorganic particles. There are some reports related to NBR-based materials’ thermal degradation and aging at the temperatures less than 200 °C [35,36,37], whereas no data on the NBR carbonization behavior was observed.

## 2. Materials and Methods

### 2.1. Materials

BNKS-18 AMN TU 38.30313-2006 nitrile butadiene rubber (NBR) (JSC Krasnoyarsk Synthetic Rubber Plant, Krasnoyarsk, Russia) with a mass fraction of acrylonitrile of 17–20 wt% was used as a matrix material. Mooney viscosity MML 1 + 4 (100 °C) for this elastomer was 42–45 units, and the ash content was 0.4 wt%.

Finely dispersed SiC powder (grade 64C, Litpromabrasiv Ltd., Moscow, Russia) was used as a filler when creating elastomeric compositions. The initial SiC powders contained mainly one carbide modification, i.e., polytype II (PSC *hP*12/14, structural type *P6.3mc*), with a lattice parameter a = 0.3081 nm. Total content of SiC in powder was not less than 98.0 wt%, and the powder contained impurities of Fe (not more than 0.4 wt%) and C (not more than 0.4 wt%).

**Figure 1:**
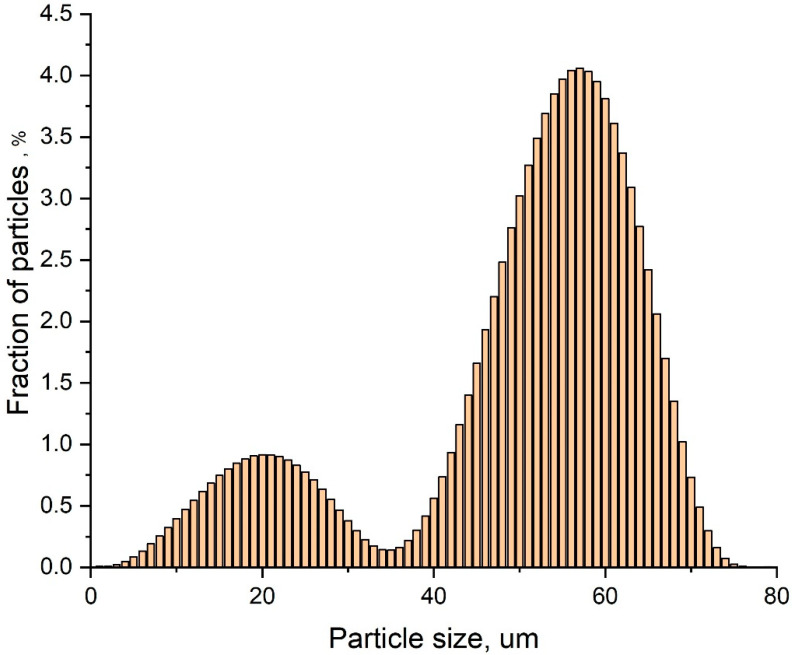
Particle size distribution of as-received SiC powder measured by laser diffraction. Bimodal behavior can be observed.

SiC particle size distribution was studied using a laser diffraction particle size analyzer Fritsch Analysette-22 Nanotech (Fritsch GmbH, Idar-Oberstein, Germany), by ISO 24235: 2007 Ceramic composites. The determination of the particle size distribution of ceramic powders by laser diffraction method is shown in Figure 1.

The SiC powder has a bimodal particle size distribution, with 20% of the particles having sizes less than 400 nm. The d10, d50 and d90 values for the filler were of 0.110, 1.93, and 4.84 µm, respectively, and the average volumetric diameter D [4.3] was 2.28 µm. The bulk density of SiC was 0.579 g/cm^3^, and the tapped density, measured by Tapped Density Analyzer—Quantachrome Instruments, in accordance with ISO 787-11, was found to be of 0.985 g/cm^3^. As will be discussed below, SiC particle size distribution affects strongly the structures and properties of composite materials.

### 2.2. Composites Formation

To study the effect of the SiC filling on the composite properties, samples containing from 0 to 450 parts per hundred rubber (PHR) with a filling step of 50 parts by weight of SiC were prepared. Sample formation includes preparing the initial components, introducing of filler and a vulcanization agent into the elastomer, vulcanization of elastomer samples and low-temperature carbonization in an inert atmosphere.

Before being added to the elastomeric mixture, SiC powder was dried at 115 °C for 6 h. The filler was introduced into NBR using laboratory rubber mixing rollers BL-6175-A (Dongguan Baopin Precision Instrument Co., Ltd., Dongguan, China). The shaft speed ratio was 1:1.25; the complete mixing and mixture homogenization time was 40 min. Dicumyl peroxide (linear formula (C_6_H_5_C(CH_3_)_2_O)_2_, CAS Number 80-43-3, Aldrich 329541, 98.0 mass. % purity, melting point of 38 °C, Sigma-Aldrich Corp., St. Louis, MO, USA) was used as a vulcanizing agent. Vulcanization of composite samples in the form of plates with dimensions of 210 × 290 × 4 mm was carried out in steel mold at a temperature of 170 °C for 10 min using an AVPM-901 vulcanization press (Tesar-Ingeneering Ltd., Saratov, Russia) with a mold constant clamping force of 5 MPa.

For the final formation of the composite samples, the obtained plates were subjected to low-temperature carbonization in an inert atmosphere (argon) with constant heating from room temperature to 340 °C for 12 h in a PM-16M muffle furnace (Electropribor LLC, Saint Petersburg, Russia). Samples for investigation were made by mechanical processing using a diamond tool.

### 2.3. Characterization of the Samples’ Structures

The obtained samples’ microstructures were investigated on thin sections and on chips prepared by cracking in liquid nitrogen using scanning electron microscopy (SEM) using a Hitachi TM-1000 microscope (Hitachi Ltd., Tokyo, Japan).

### 2.4. Density Measurements

The densities of vulcanized and carbonized samples were measured by hydrostatic weighing in distilled water and ethyl alcohol according to ISO 1183-1: 2019 (Plastics—Methods for determining the density of non-cellular plastics) using an AND GR 202 analytical balance (A & D Limited, Tokyo, Japan) equipped with a hydrostatic weighing AD-1653.

### 2.5. Hardness Measurements

The hardnesses of vulcanized samples on the Shore A scale and of carbonized samples on the Shore D scale were measured using TSh-A and TSh-D handheld durometers (Novotest, Saint Petersburg, Russia) in accordance with ISO 868:2003 (Plastics and ebonite; determination of indentation hardness using a durometer (Shore hardness)).

### 2.6. Mechanical Tests

Three-point bending and compression tests were carried out at room temperature using Zwick/Roell Z020 universal tensile testing machine (Zwick GmbH, Ulm, Germany) using a MultiXtens high-precision strain measurement system. Before testing all the samples were conditioned in accordance with ISO 291: 2008 (Plastics—Standard atmospheres for conditioning and testing) under a standard 23/50 atmosphere for 88 h. The total number of specimens tested at one point in compression and three-point bending tests was not less than 7.

Flexural tests were carried out in accordance with ISO 178: 2019 (Plastics—Determination of flexural properties); the studied samples were 80 × 10 × 4 mm. The samples were loaded until fracture at a test speed of 2 mm/min.

Compression tests of carbonized samples were performed according to ISO 604:2002 (Plastics—Determination of compressive properties). The breaking force was measured using specimens with dimensions of 10 × 10 × 4 mm, and elastic modulus was measured using samples with dimensions of 50 × 10 × 4 mm. The test speed was 2 mm/min. To obtain elastic modulus value the load was applied to the samples while taking into account the required correction of the initial part of strain–stress plot in accordance with ISO 604:2002.

### 2.7. Thermal Conductivity Investigation

Thermal diffusivity was measured in the temperature range from 25 to 300 °C in accordance with ASTM E1461-07 (Standard Test Method for Thermal Diffusivity by the Flash Method) using the NETZSCH LFA447 NanoFlash device (Netzscg GmbH, Selb, Germany). The study was carried out using cylindrical specimens each having a 12.7 mm diameter and a thickness of 1–1.5 mm.

Specific heat capacities C_p_ of composite materials in the temperature range from 25 to 300 °C were measured using a NETZSCH DSC 204 Phoenix F1 differential scanning calorimeter (Netzscg GmbH, Selb, Germany) in accordance with ISO 11357-4: 2014 (Plastics—Differential scanning calorimetry (DSC)—Part 4: Determination of specific heat capacity). Sapphire was used as standard reference. The tests were carried out on pieces 5 mm in diameter and weighing 24–25 mg, in a protective argon atmosphere.

Thermal conductivity was calculated using formula:

(1) $$\lambda \left(t\right)=a\left(t\right)\cdot d_{k}\cdot C_{P}\left(t\right)$$

where *λ*(*t*) is thermal conductivity coefficient at a certain temperature t, W/(m·K); *a*(*t*) is thermal diffusivity at certain temperature t, mm^2^/s; *d_k_* is material density, g/cm^3^; *C_p_*(*t*) is specific heat capacity, J/(g·K).

### 2.8. Thermogravimetric Analysis

The study of the thermodynamic stability of vulcanized and carbonized samples was performed by thermogravimetric analysis (TGA) using a TGA Q500 TA Instruments thermogravimetric analyzer (TA Instruments, New Castle, DE, USA). The study was carried out in an atmosphere of an inert gas (argon, purity 99.998%) in the mode of heating to a temperature of 600 °C at a rate of 5 K/min, followed by isothermal holding.

## 3. Results and Discussion

**Figure 2:**
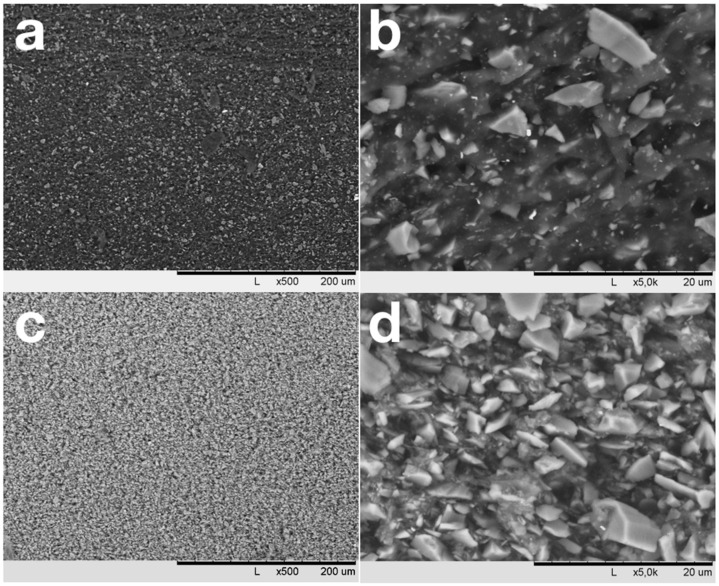
SEM images of vulcanized samples with filling degrees of 50 (**a**,**b**) and 450 (**c**,**d**) PHR.

Figure 2a,c shows that mixing in rollers results in a homogeneous distribution of SiC over the NBR matrix. SiC particles with sizes less than 1–1.5 µm are well wetted with NBR and are immersed in the elastomer (Figure 2b). In contrast, coarse, non-equiaxed SiC particles are weakly wetted by NBR and crumble from the cut surface. At high degrees of filling (Figure 2c), the NBR amount is not enough for the elastomer to provide an interlayer between the NBR particles, and pores with sizes up to 0.5 μm appear in the material between coarse filler particles. Observed SEM images allow one to conclude that NBR/SiC interaction is strongly affected by SiC particle size. Thereafter, significant influences of the observed bimodal distribution of SiC particles size (Figure 1) on the composites’ properties can be expected.

**Figure 3:**
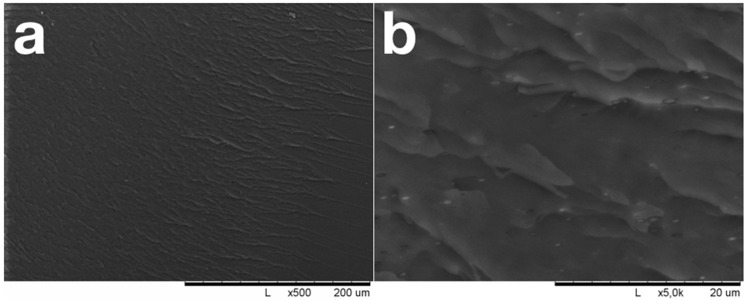
SEM images of carbonized pure NBR fracture surface.

During low-temperature carbonization of unfilled NBR, a homogeneous, weakly defective black material is formed (Figure 3); the density of the unfilled NBR increases from 0.94 g/cm^3^ to 1.07 g/cm^3^ during carbonization. As a result of the carbonization, vulcanized NBR loses its ductility and breaks down brittlely under load. The fracture surface was nearly smooth and contained no defects or gas bubbles. Brittle crack evolution steps are clearly visible on the fracture surface.

**Figure 4:**
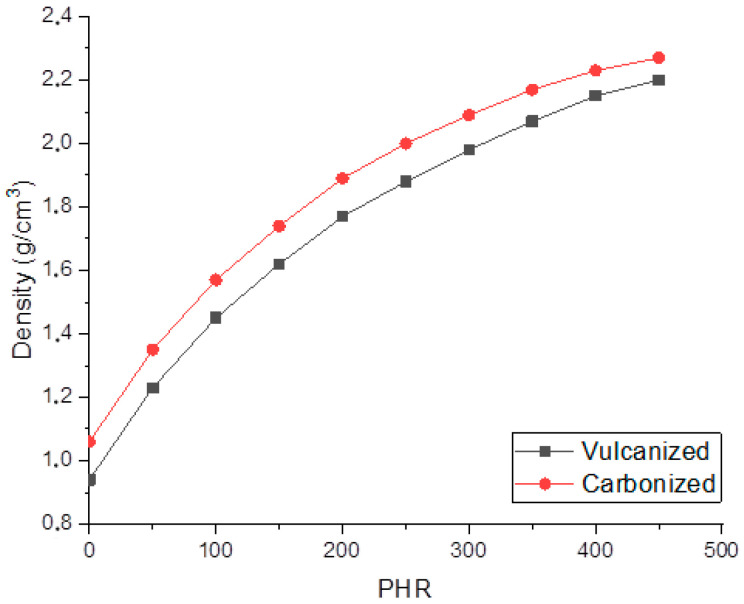
Dependencies of vulcanized and carbonized composites density on the filling degree.

Figure 4 shows that the concentration dependencies of both vulcanized and carbonized composites’ densities are non-linear. The growth rate of the density magnitude decreases with an increase in the filling degree. At low degrees of filling, sample density increases by 0.13–0.12 g/cm^3^ with an increase in the degree of filling by 50 PHR, whereas at a high the degree of filling, the rise in density with an increase in the degree of filling by 50 PHR decreases to 0.07–0.08 g/cm^3^. Evaluation of the porosity was carried out on the basis of pure matrix and silicon carbide (3.21 g/cm^3^) densities using the additive law. The porosity of vulcanized composites varied from 26 to 19.5%; carbonization results in the shrinkage of the matrix, and the porosity decreases by 3.5–4% as a result of carbonization. SEM data allows one to propose that pores formed in the material are meso-pores and micro-pores with sizes less than 500 nm. Obtained values of density and porosity are summarized in Table 1.

**Figure 5:**
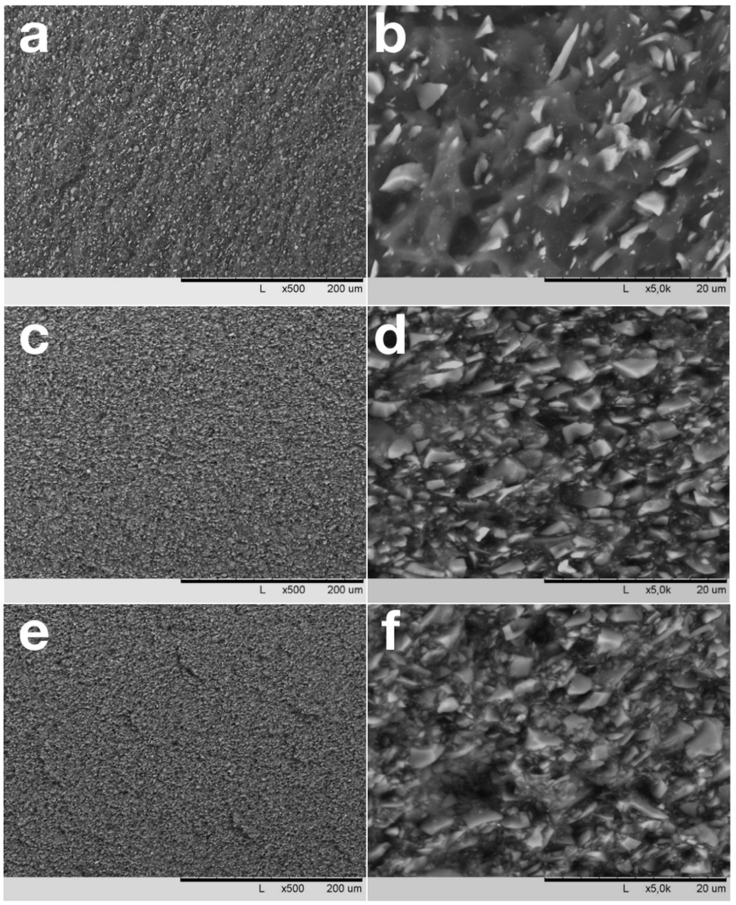
SEM images of carbonized samples with filling degrees of 50 (**a**,**b**), 250 (**c**,**d**) and 450 PHR.

Low-temperature carbonization of composites results in the formation of fine-grained material, consisting of homogeneously distributed SiC particles interconnected by interlayers of thermally transformed NBR matrix (Figure 5). At low degrees of filling due to the homogeneous distribution of fillers in matrix, there are nearly no contacts between SiC particles (Figure 5b). At a filling degree of 200–250 PHR, SiC particles form a continuous network due to contacts between neighboring filler particles (Figure 5d). At the highest degrees of filling (400–450 PHR), the matrix interlayers between the particles become thin, and the amount of matrix material in this case is not enough to cover the surfaces of all particles completely (Figure 5f).

**Figure 6:**
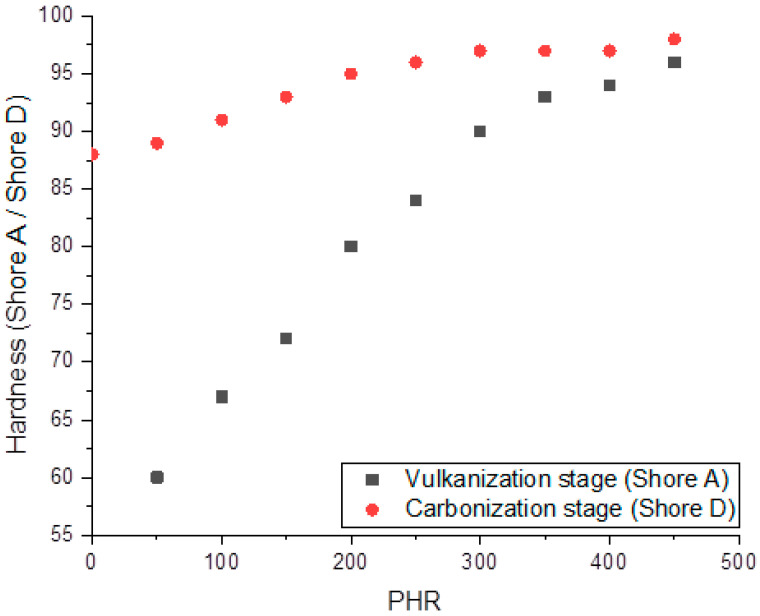
Dependencies of Shore A (for vulcanized composites) and Shore D (for carbonized composites) hardness on the filling degree.

Figure 6 shows the concentration dependencies of vulcanized and carbonized composites’ hardnesses. The hardness of vulcanized specimens increases almost linearly from 60 to 90 Shore A units in the range of filling degree up to 300 PHR, and further increases in filler content have nearly no effect on the hardness. The hardness of carbonized samples, measured on the Shore D scale, increases linearly up to a filling of 200 PHR; further increases in PHR hardness magnitudes cause nearly no change. Weak concentration dependence of hardness at high PHR may be associated with the formation of a network of mutually contacting SiC particles with sizes of 5–10 µm.

**Figure 7:**
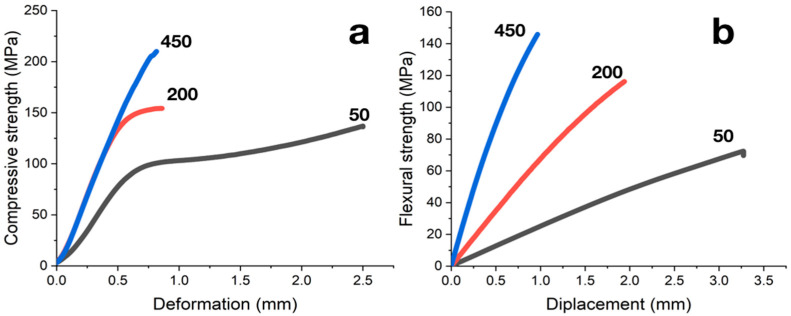
(**a**) Strain–stress diagrams from compression tests of carbonized samples; filling degree in PHR is indicated near curves. (**b**) Load-deflection diagrams from flexural tests of carbonized samples; filling degree in PHR indicated near curves.

Failure of carbonized samples during compression tests occurs along shear planes lying at angles of 45° to the load application direction. The shape of a strain–stress diagram depends on the composite’s filling degree (Figure 7a). Samples with filling of less than 100 PHR have stress–strain curves typical for semi-rigid filled compositions (curve type a according to ISO 604:2002). Increasing in filling degree results in the increase in the rigidity of the composites; at degrees of filling more than 300 PHR compression behavior is typical for rigid materials, and strain–stress curves correspond to type b according to ISO 604:2002.

**Figure 8:**
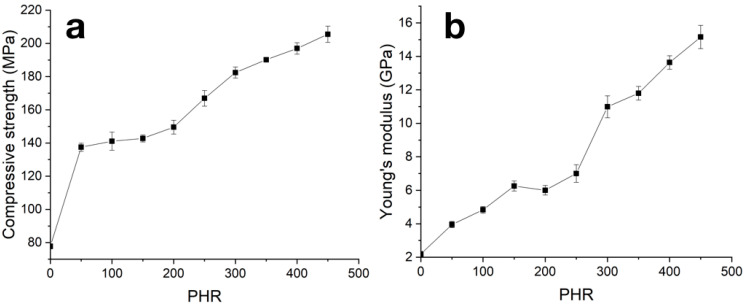
Concentration dependencies of strength (**a**) and Young modulus (**b**) from compression tests of carbonized samples.

With an increase in the SiC content in the material, both the Young’s modulus and compressive strength increase (see Figure 8 and Table 1). Observed concentration dependencies can be divided into three stages with different rates of the modulus and strength magnitude increases: initial from 0 to 200 PHR, average from 200 to 300 PHR and final at filling degree more than 300 PHR. Transition from the initial stage to the average one may be evidence of the formation of a network by coarse SiC particles at degrees of filling over 200 PHR; transition from the average stage to the final stage can occur due to insufficiency of the matrix material amount at high filling degrees. Thereafter, at the initial stage, increases in mechanical properties with an increase in PHR can be associated with increases in matrix body and boundary layer strength, due to their filling with isolated SiC particles. In the average stage, formation of the contact net became the main driving force of mechanical property growth, and in the final stage, when dense contact between SiC particles was formed, increases in mechanical properties were provided by a decrease in residual matrix material amount.

The load–deflection diagrams obtained from flexural tests of composites are weakly nonlinear (Figure 7b). The failure of samples, regardless of the degree of filling, occurred instantly when maximum stresses were reached; specimens were divided into 2 or 3 fragments.

**Figure 9:**
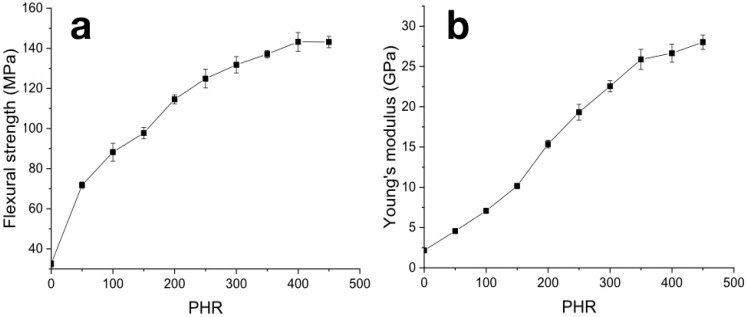
Concentration dependencies of strength (**a**) and Young modulus (**b**) from flexural tests of carbonized samples.

The flexural strength increased from 32 MPa for pure carbonized NBR to 140 MPa for samples filled with 400 PHR (Figure 9a). A further increase in filling degree up to 450 PHR results in no change in flexural strength. Changes in the growth rate of flexural strength are not as pronounced as in cases of compression tests. However, a weakly pronounced reduction in the strength growth rate was observed at a degree of filling of 200 PHR. The flexural modulus increased over the entire concentration range (Figure 9b) from 2.1 GPa for pure canonized NBR to 27.7 GPa for maximum filling degree of 450 PHR. The rate of elasticity modulus growth decreases at degrees of filling above 350 PHR.

**Figure 10:**
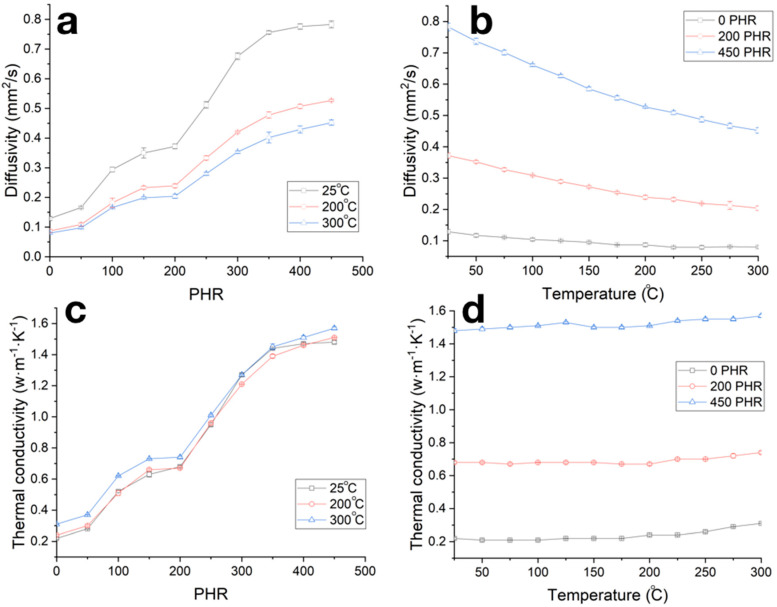
Dependencies of thermal diffusivity (**a**,**b**) and thermal conductivity coefficient (**c**,**d**) on the filling degree (**a**,**c**) and temperature (**b**,**d**) for carbonized composites.

Concentration dependencies of the carbonized samples’ thermal diffusivities (Figure 10a and Table 1), along with in the case of compressive strength, can be divided into three stages differing in the rate of thermal diffusivity increase. An increase in the measurement temperatures in the range of 25–300 °C resulted in the decrease in thermal diffusivity by 25–30% (Figure 10b). Concentration and temperature dependencies of thermal conductivity coefficient calculated by formula (1) are given in Figure 10c,d; see also Table 1. The thermal conductivity of the obtained composites slightly depends on the temperature, whereas increase in the filling degree results in the increase in thermal conductivity coefficient from ~0.2 W/(m·K) for unfilled sample to ~1.6 W/(m·K) for samples with filling degree of 450 PHR.

## 4. Summary and Conclusions

Silicon carbide is well dispersed and evenly distributed in the NBR matrix when using mixing rollers. Particles with sizes less than 1–1.5 µm are well wetted with rubber and are usually located inside the matrix material at fractures Figure 2b and Figure 5b). It allows us to assume that for SiC in a vulcanized or carbonized NBR matrix the critical size of filler particles is close to 1–1.5 μm. The observed difference allows one to suggest that the heat dissipation at the filler–matrix interfaces will depend on the SiC particles sizes. The number of chipping particles on samples’ fracture surfaces decreases in carbonized samples compared to vulcanized ones, which shows that the adhesive interaction between NBR and SiC increases due to carbonization. An increase in the adhesion between matrix and fillers well explains the significant increases in the elastic and strength characteristics of carbonized materials in relation to vulcanized samples. As the proportion of SiC particles with sizes less than 1 μm was about 35% of the total, such particles can effectively change the properties of not only a thin interfacial layer but also the entire volume of the matrix, changing its strength and deformation characteristics.

The change in the properties of carbonized materials with an increase in the degree of filling can be affected by two factors. The effects of particles with sizes less than critical consist of a decrease in plasticity and an increase in the strength of the matrix, whereas SiC particles with dimensions more than critical (1.5 μm) create fields of local stresses and microcracks in the material, which enable energy dissipation of macrocracks during the fractures process.

**Figure 11:**
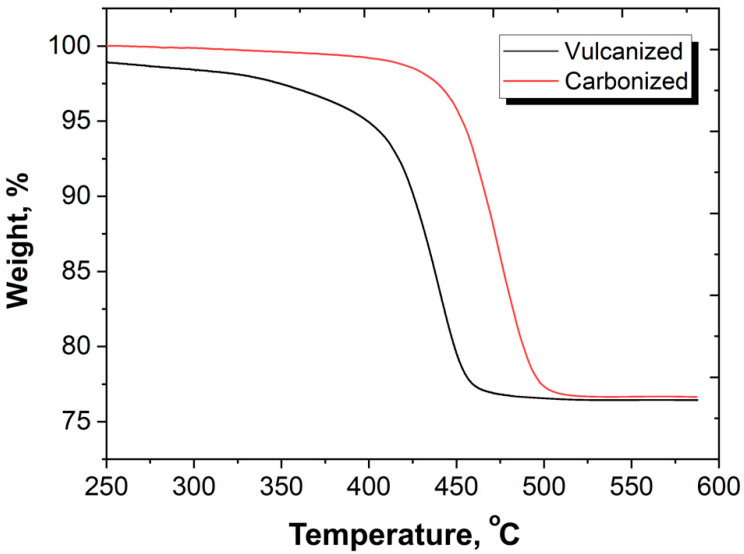
TGA curves of vulcanized and carbonized samples with SiC of 200 PHR.

In the process of thermal degradation and low-temperature carbonization, the NBR used to form the composite matrix undergoes profound changes, resulting in the formation of a homogeneous, nearly defective-less black material (Figure 3). Carbonization results in significant changes in the processes of deformation and destruction, in thermal properties and in the thermal resistances of materials. Figure 11 illustrated the increase in thermal resistance in the low-temperature carbonized composites compared with the vulcanized one. It is seen that for vulcanized sample, weight loss begins at temperatures of 180–200 °C. At temperatures of 320–330 °C, the weight of vulcanized composites starts to decrease drastically. Such behavior is typical for NBR and NBR bases composites [38,39]. As a result of low-temperature carbonization, the thermal stability of the material increases significantly. For carbonized samples the change in mass up to a temperature of 400 °C in an inert atmosphere does not exceed 1 wt%; intense destruction of carbonized samples begins at temperatures above 430 °C.

The weight loss during carbonization is 10% due to the removal of H_2_, N_2_, NO, CO_2_ and H_2_O by evaporation, and the relative amount of carbon in the material increases [25]. Carbonization is accompanied by material shrinkage; as a result, the density of NBR increases from 0.94 to 1.07 g/cm^3^ (see Table 1). The beginning of intensive thermo-oxidative destruction at carbonization for NBR based materials lies at a temperature of more than 400 °C [26], which is higher than the temperature used for carbonization in the present study.

As a result of low-temperature carbonization, the hardness of pure NBR increased from 58 Shore A to 88 Shore D, whereas the plasticity of the material significantly reduced. Yield stress at compression for pure NBR after carbonization increased from 3–4 MPa to 72 MPa, and fracture occurred at stress of 128 MPa. Youngs’ modulus increased by two orders of magnitude from 20 MPa for vulcanized to 2.1 GPa for carbonized NBR.

Filling with SiC results in significant increases in compressive strength and Young modulus; at a SiC content of 450 PRH these magnitudes are 212 MPa and 15.1 GPa, respectively. However, based on of flexural tests, the filling degree of 400 PRH can be considered as critical, and a further increase in filling degree results in decreases in the strength characteristics of the material. The obtained materials in terms of their strength characteristics are superior to the phenol formaldehyde resin carbonized under similar conditions [40]—for which the flexural strength is less than 80 MPa at an elastic modulus is of 5 GPa—and are not inferior to modern reinforced engineering plastics [1,2,22].

Thermal conductivity of NBR during carbonization does not change and is about 0.2 W/(m·K), which is a typical value for a significant number of synthetic rubbers and polymer materials [1,2,3,41]. The increase in the thermal conductivity of the composite material is due to the alignment of optimal heat transit paths through the filler net [2,3,42]. In our study a significant increase in thermal conductivity was observed in two filling degree ranges of 50–100 and 200–350 PRH (Figure 10a,c). As is shown in Figure 5d, at a PRH of 200 and higher, coarse SiC particles form a contacting heat-transfer net, and the density of this net increases up to 350 PRH, which leads to an increase in thermal conductivity from 0.75 to 1.6 W/(m·K) with an increase in PRH from 200 to 350 PRH. The observed increase in thermal conductivity was from 0.2 to 0.7 W/(m·K) at filling degree 50–100 PHR, at which point the conductive net was not yet formed; that can be explained by increases in the thermal conductivity of the interfacial layer and matrix volume, due to the level of adhesion to the NBR matrix from the coarse SiC particles that did differ critically in size.

We can conclude that the properties of carbonized materials are characterized by three filling degrees: (1) the stage up to 200 PRH; (2) the stage of 200–350 PRH within which the most significant increase in material characteristics is observed; (3) the section with a filling degree of more than 350 PRH, at which point the increases in properties tend to terminate.

Evaluation of the porosity of the obtained samples based on the density of the carbonized matrix (1.07 g/cm^3^) and the density of silicon carbide (3.21 g/cm^3^) gave values in the range of 30–19.5%, and decreases with increasing degree of filling—that formed pores were meso- and micropores with sizes less than 500 nm. In the process of carbonization of vulcanized samples (Table 1) at filler concentrations up to 300 PRH, the porosity decreased by 3.5%, and the density of the samples increased by a constant value of 0.12 g/cm^3^. At higher SiC contents, the increase in density, and correspondingly, the decrease in porosity slows down, which suggests that additional nets of cracks and micropores remain in the material after carbonization. The presence of such defects leads to decreases in the characteristics of highly filled composites.

Thus, we conclude that the optimal degree of filling for composite materials based on carbonized NBR matrices is 300–350 PRH, at which point the maximum increase in the entire complex of properties is achieved. The combination of the high hardness, compressive and flexural strength; high thermal conductivity; and high heat resistance of such materials [25,26] makes it possible to consider the obtained composites as promising for creating mechanical seal systems operating in corrosive environments containing abrasive particles.

## Figures and Tables

**Table 1:** Density, porosity, mechanical and thermal properties of NBP-based composites. Mechanical and thermal properties are given for carbonized samples.

PHR	Density, g/cm^3^		Porosity, %		Compression Test		Flexural Test		Thermal Diffiusivity, mm^2^/s		Thermal Conductivity, W/m·K	
	Vulc	Carb	Vulc	Carb	σ, MPa	Е, GPa	σ, MPa	Е, GPa	25 °C	300 °C	25 °C	300 °C
0	0.94	1.07	-	-	77.8 ± 4.4	2.2 ± 0.14	32.6 ± 1.0	2.16 ± 0.14	0.129 ± 0.02	0.08 ± 0.02	0.22 ± 0.01	0.31 ± 0.01
50	1.23	1.35	27.50	24.3	137.4 ± 2.4	3.95 ± 0.20	71.8 ± 1.6	4.56 ± 0.12	0.166 ± 0.02	0.098 ± 0.02	0.28 ± 0.01	0.37 ± 0.01
100	1.45	1.57	30.12	26.64	141.1 ± 9.5	4.83 ± 0.20	88.2 ± 4.4	7.07 ± 0.22	0.294 ± 0.03	0.166 ± 0.02	0.52 ± 0.02	0.62 ± 0.01
150	1.62	1.74	29.63	26.08	144.3 ± 0.7	6.26 ± 0.30	97.7 ± 2.9	10.15 ± 0.25	0.35 ± 0.03	0.199 ± 0.02	0.63 ± 0.02	0.73 ± 0.02
200	1.77	1.89	27.85	24.3	153.0 ± 1.8	6.00 ± 0.28	114.5 ± 2.2	15.36 ± 0.48	0.372 ± 0.04	0.204 ± 0.03	0.68 ± 0.01	0.74 ± 0.01
250	1.88	2.00	26.6	23.03	166.9 ± 4.7	7.00 ± 0.53	124.9 ± 4.6	19.31 ± 0.98	0.512 ± 0.02	0.28 ± 0.01	0.95 ± 0.01	1.01 ± 0.02
300	1.98	2.09	25.07	21.87	184.1 ± 1.2	11.00 ± 0.41	131.8 ± 4.1	22.54 ± 0.69	0.676 ± 0.03	0.353 ± 0.02	1.27 ± 0.02	1.27 ± 0.01
350	2.07	2.17	23.49	20.64	190.2 ± 0.4	11.8 ± 0.43	137.1 ± 1.9	25.87 ± 1.25	0.756 ± 0.03	0.402 ± 0.04	1.44 ± 0.01	1.45 ± 0.01
400	2.15	2.23	21.99	19.84	197.0 ± 3.4	13.64 ± 0.40	143.2 ± 4.8	26.64 ± 1.12	0.776 ± 0.02	0.429 ± 0.02	1.47 ± 0.01	1.51 ± 0.01
450	2.20	2.27	21.35	19.53	205.5 ± 6.9	15.16 ± 0.71	143.1 ± 2.8	28.01 ± 0.89	0.783 ± 0.03	0.452 ± 0.03	1.48 ± 0.02	1.57 ± 0.01
